# Nontuberculous mycobacteria in gastrostomy fed patients with cystic fibrosis

**DOI:** 10.1038/srep46546

**Published:** 2017-04-24

**Authors:** H. Al-momani, A. Perry, R. Jones, S. Bourke, S. Doe, J. Perry, A. Anderson, T. Forrest, I. Forrest, M. Griffin, M. Brodlie, J. Pearson, C. Ward

**Affiliations:** 1 Institutes of Cellular Medicine and Cell & Molecular Biosciences, Newcastle University Medical School, Newcastle University, Newcastle upon Tyne, NE2 4HH, UK; 2 Department of Microbiology, Newcastle upon Tyne Hospitals NHS Foundation Trust, Freeman Hospital, Newcastle upon Tyne, NE7 7DN, UK; 3 Adult Cystic Fibrosis Centre and Northern Oesophago-Gastric Unit Royal Victoria Infirmary, Newcastle upon Tyne, NE1 4LP, UK

## Abstract

Multi-drug resistant Mycobacterium abscessus complex (MABSC) is a form of Nontuberculous mycobacteria (NTM) of special, international concern in Cystic Fibrosis (CF). We hypothesised that gastric juice and percutaneous endoscopic gastrostomy (PEG) feeding devices might yield MABSC isolates. Gastric juice and sputa from sixteen adult PEG fed CF patients and five replaced PEG tubes were studied. Bacterial and fungal isolates were cultured. Mycobacterium were identified by rpoB, sodA and hsp65 gene sequencing and strain typed using variable number tandem repeat. Bacteria and/or fungi grew from all gastric juice, sputa and PEG samples. MABSC were detected in 7 patients. Five had MABSC in their sputum. Two had an identical MABSC strain in their sputum and gastric juice and one had the same strain isolated from their PEG tube and sputum. Two patients who were sputum sample negative had MABSC isolated in their gastric juice or PEG tube. MABSC were therefore identified for the first time from a gastric sample in a minority of patients. We conclude that gastric juice and PEG-tubes may be a potential source of MABSC isolates in CF patients, and these findings warrant further study.

Non tuberculous mycobacteria (NTM) are important human pathogens responsible for a wide spectrum of soft tissue infections, which are recognised to cause disseminated infection in immunocompromised patients. The incidence and prevalence of pulmonary NTM infection is increasing worldwide, contributing to concerns that NTM infection may become a serious health challenge^1^.

NTM infection is of special, international concern in people with CF and represents a significant emerging threat in patients with CF. Estimates of the prevalence of NTM in the CF population range from 1.3% to 32.7% depending on the geographical region and patient population^2^. To date, the largest CF studies report rates of NTM-positive respiratory cultures between 6.6% and 13.7%^2^.

Of the rapidly growing NTM species, the multidrug resistant *Mycobacterium abscessus complex* (MABSC) has emerged as a major respiratory pathogen particularly in individuals with CF, that is associated with an accelerated decline in lung function^3^. MABSC can also prevent safe lung transplantation, which is one of the few therapeutic approaches available in end stage lung disease. It is for this reason that many transplant centres consider active MABSC pulmonary disease to be a relative contraindication to lung transplant listing^4^. Treatment of MABSC is challenging, requiring extended therapy with combinations of multiple antibiotics required that may be associated with adverse effects and is not always successful clinically^5^.

MABSC can be divided into three subspecies (subsp): *Mycobacterium abscessus subsp abscessus, M abscessus subsp massiliense*, and *M abscessus subsp bolletii*^6^. A recent publication demonstrated a potential high rate of transmission of MABSC between CF patients despite stringent segregation^7^. It was postulated that transmission could occur indirectly, through fomite contamination or through aerosol generated during physiotherapy and spirometry testing^8^. The study also demonstrated that inferred transmission might occur from patients with persistent smear negative, culture positive sputa. This suggested that only a low level of inoculum might be needed for infection, contributing to debate regarding the need for rigorous preventative measures^8^.

We have recently described findings of a gastric juice reservoir of organisms relevant to CF lung pathophysiology in patients fed by percutaneous endoscopic gastrostomy (PEG) tubes, which also confirmed a widespread presence of extra oesophageal reflux (EOR) symptoms and potential for micro-aspiration in patients with CF^9^,^10^.

This is consistent with the established diagnostic role for gastric aspirate culture in pulmonary tuberculosis^11^,^12^ and the finding that repeated gastric lavage has better sensitivity for microbiological diagnosis of childhood pulmonary tuberculosis compared with bronchoalveolar lavage^11^,^13^. The potential gastric juice culture of MABSC in people with CF has not been established to our knowledge however.

We hypothesised that gastric juice and PEG tubes from people with CF might yield NTM isolates, and in particularly MABSC. The aim of this cross-sectional study was therefore to provide a description of the NTM isolates of sputum, gastric juice and PEG tubes from adult CF patients.

## Methods

### Ethical approval

Research Approval was obtained from Newcastle and North Tyneside Research Ethics Committee to perform research on samples collected as part of the Hepatopancreatobiliary groups biobank based in Newcastle University. All study participants provided written informed consent prior to initiation of the study. All methods were carried out in accordance with relevant guidelines and regulations.

### Patients and sample

CF patients receiving PEG feeding represented an important opportunity to directly sample gastric juice, with less potential for contamination with oropharnygeal commensals. From the 270 CF patients attending the regional CF Centre, a potential 16 PEG-tube fed patients were available for study (Table 1).

Following overnight fasting gastric juice and sputum samples from those PEG-tube fed CF patients (CF 1–16). PEG tubes which became available due to renewal or removal as part of routine PEG management were collected form 5 patients (CF 4–6 and CF 14–15). All the potentially available patients with PEG tubes in the region were therefore included in this study. No PEG fed CF patients were excluded or positively selected for in the study.

### Symptoms of extraoesophageal reflux

Patients with CF had symptoms of extraoesophageal reflux (EOR) assessed using the Reflux Symptoms Index (RSI) score; with a score of less than 13 classed as not EOR symptomatic^14^. The reflux symptom index is a patient reported outcome measure designed to assess laryngeal symptoms secondary to reflux, including cough.

### Sample processing

Gastric juice pH was measured using pH strips and sputum samples were homogenised with equal amounts of Dithiothreitol for 1 to 3 minutes. The inner and outer part of the PEG tube were divided into small pieces and vigorously washed with 3 ml saline, yielding a PEG conditioned saline (PEG-s).

### Microbiological culture conditions

Gastric juice, sputum samples and PEG-s underwent microbiological culture performed in accordance with national accredited methods. 10 μl of homogenised sputum, gastric juice and PEG-conditioned saline were plated for bacterial and fungal cultures. The following media were used: Columbia blood agar supplemented with 5% horse blood, chocolate agar supplemented with 70 mg/L bacitracin, Burkholderia selective agar (for CF sputa and gastric juice, CEP bioMérieux UK), cysteine lactose electrolyte deficient agar (CLED) and fastidious anaerobic agar (FAA). Plates were incubated according to a standard protocol, CEP cultures were incubated for 10 days at 30 °C for isolation of Burkholderia cepacia complex and rapidly growing mycobacterium.

Plates were examined daily for evidence of microbial growth and assessment of the number of distinct colonial variants was recorded. All morphological variants were sub-cultured and used for identification and stored at −20 °C in 10% glycerol skimmed milk. Isolates were identified by matrix assisted laser desorption ionisation time-of-flight (MALDI-TOF) mass spectrometry (Bruker Daltonics, UK) and where necessary, appropriate API kits (bioMérieux, UK)^15^. Mycobacterium was identified by rpoB, sodA and hsp65 gene sequencing and strain typed using variable number tandem repeat (VNTR), Colindale, UK^7^.

### Statistical analysis

Univariate analysis was used to compare differences in demographic and clinical variables between CF patients with and without MABSC isolates. Comparisons were unpaired, and all tests of significance were two-tailed.

All data analyses were performed using SPSS for Windows, version 22; p-values < 0.05 were considered to be statistically significant.

## Results

### CF Patients

This patient cohort had moderate to severe CF lung disease, which was in keeping with this PEG fed population (median FEV1, 1.6 L (37% predicted) range 0.5–2.7 L (17–88%), along with as is the norm in patients with CF, long term antibiotic exposure and use of acid suppression medication (Table 1).

### Sputa and gastric juice microbiological results

**Figure 1:**
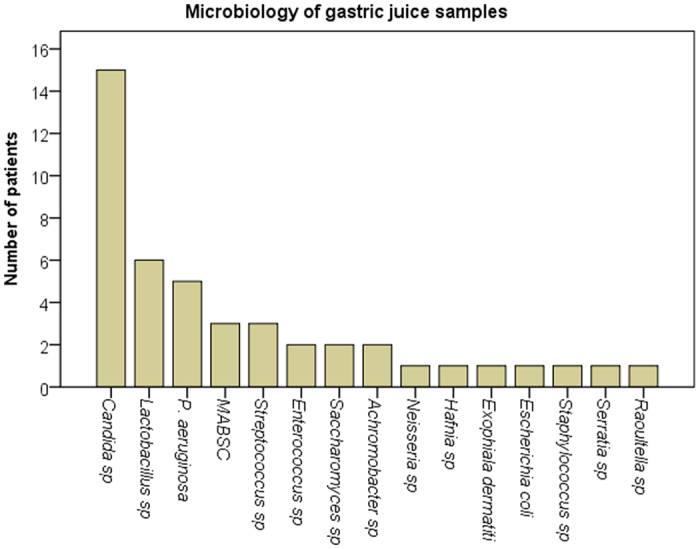
Microbiology culture results for CF gastric juice samples.

Of the 16 patients investigated, the most frequent microorganisms detected were *Streptococcus* spp, MABSC, *P. aeruginosa* and *Candida* spp in both sputum and gastric juice samples. The exceptions were *Aspergillus* and *Rothia* spp which were more common in sputum samples while *lactobacillus* spp was more common in gastric juice samples (See e-Tables 1 and 2 in the online data supplement for CF gastric juice and CF sputum samples culture results). The most frequently isolated bacterial taxa from the gastric juices were *Lactobacillus* (6/16), *P. aeruginosa* (4/16), *Streptococcus* spp (3/16), and MABSC (3/16). *Candida* species were isolated from 14 gastric juice samples (Fig. 1).

**Figure 2:**
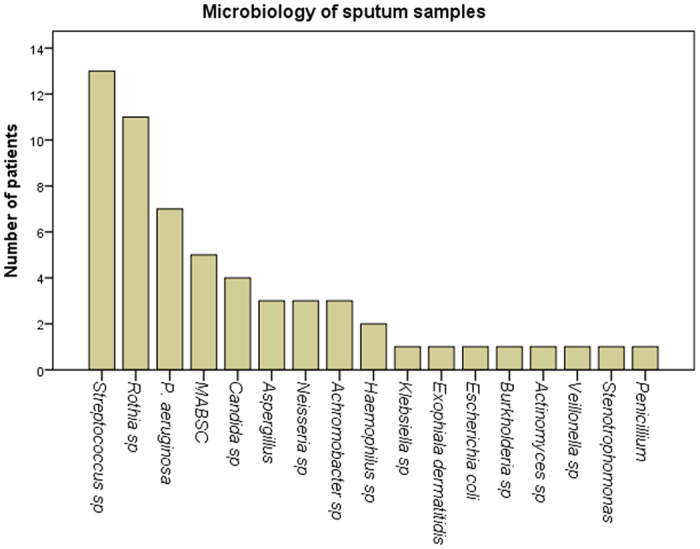
Microbiology culture results for CF sputum samples.

Identification of the bacteria isolated from 16 sputum samples revealed 13 different bacterial genera with *Streptococcus* spp (13/16), *P. aeruginosa* (7/16), MABSC (4/15), *Achromobacter* spp (3/16) and *Rothia* (11/16) the most frequently isolated from sputum samples. *Candida* species only were isolated in 4 sputum samples (Fig. 2).

### Microbiological findings in PEG tube conditioned saline (PEG-s)

**Figure 3:**
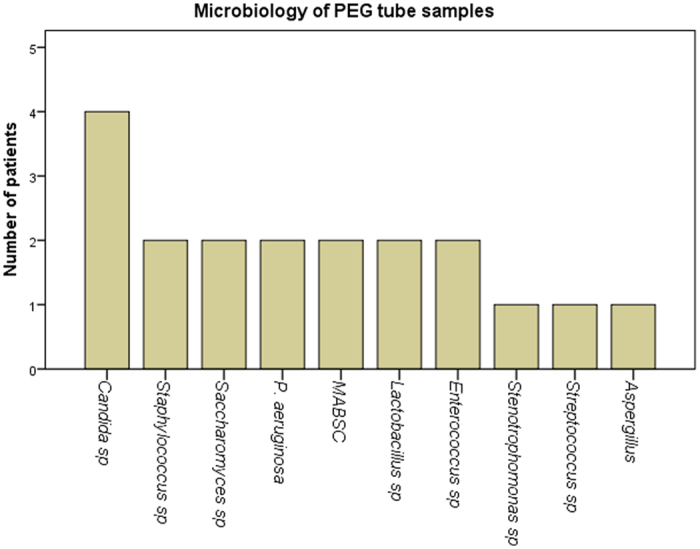
Microbiology culture results for percutaneous endoscopic gastrostomy tube conditioned saline (PEG-s) samples obtained from people with CF.

PEG tubes were removed from 5 patients (CF4-6 and CF-14-15) as part of their routine management, not due to clinical suspicion regarding potential microbiological infection. Bacterial and fungal species were isolated from all PEG tubes. The bacterial species isolated included MABSC, *P. aeruginosa, lactobacillus, Enterococcus* and *Staphylococcus sp*, isolated in 2 patients. *Candida* species were isolated in 4 patients (Fig. 3).

### MABSC culture results

MABSC were detected in 7 out of 16 CF patient (43%), 5 patients had MABSC in their sputum, 3 patient with *Mycobacterium massiliense* (CF-3, CF-9, CF-13) and 2 with *Mycobacterium abscessus sub sp abscessus* (CF-6 and CF-4). Among the 5 patients with sputum isolates, 2 had the identical strain of MABSC confirmed by VNTR in their gastric juice (*M. massiliense* in CF-3 and CF-13) and one patient had the same strain isolated from their PEG tube (*M. abscessus sub sp abscessus* (CF-4).

2 patients who were sputum sample negative for MABSC (CF-1 and CF-14) had either *M. massiliense* in their gastric juice (CF-1) or *M. abscessus sub sp abscessus* and *M. bolletii* isolated from their PEG tube (CF-14). MABSC were therefore isolated for the first time from a non-respiratory lung, gastric site, in these patients (Table 2).

### Longitudinal sputum MABSC culture results in CF patients where MABSC was first isolated from a non-respiratory, gastric site

MABSC was first isolated from a non-respiratory, gastric site in CF patients 1 and 14. Negative sputum samples were obtained through the clinical surveillance samples obtained at clinic visits prior to isolation of NTM from the gastric compartment.

CF 1. *M. massiliense* was isolated from gastric juice on 23-1-15. Eighteen sputum samples, over 20 months, were examined for NTM prior to this (dates: 22-5-13 to 23-1-15). Following the positive gastric sample, ten sputa were negative for NTM after this (23-1-15 to 2-3-16). A transient isolation of sputum *M. abscessus subspecies abscessus* occurred on 2-3-16. Seven subsequent sputa were negative for NTM (21-3-16 to 2-12-16).

CF 14. The positive gastric sample (percutaneous endoscopic gastrostomy tube) was identified on 15-7-14, with isolates of *M. abscessus subspecies abscessus* sequence type 26 and *M. bolletti*. Prior to this five lung samples were examined for NTM (9-8-13 to 26-6-14; two sputa and three cough swabs). Seventeen subsequent sputum samples were examined and were negative for NTM (dates 13-8-14 to 22-10-16).

The longitudinal culture data were therefore consistent with the possibility that a gastric sample can represent the first isolation of NTM in people with CF independent of sputum samples.

### Symptoms of extraoesophageal reflux in CF patients

Symptom scores for extraoesophageal reflux were available in 14 of the 16 patients with CF (Table 1). 13 patients were EOR symptomatic, despite the use of PPI or Ranitidine, with an RSI score >12; median RSI score 17 (range 13–36). One patient was EOR non symptomatic with RSI score 8.

RSI scores for patients positive for MABSC were higher (median 22, range 17–36) compared to patient who were negative for MABSC (median 15, range 8–20), but this was not statistically significant (p = 0.22). The cough domain score of the RSI questionnaire in MABSC positive patients ranged from 2–5, with 5 the highest score possible.

## Discussion

For the first time to our knowledge, we have shown that MABSC can be isolated from the gastric juice and PEG tubes of patients with CF in addition to sputum. Previous reports where gastric juice has been shown to be an advantageous sample for diagnosing Mycobacterium tuberculosis add plausibility to this main finding^11^,^12^. We also found that isolates were obtained from a gastric source with MABSC negative sputa. MABSC were therefore identified for the first time from a gastric sample in these patients, despite rigorous and extended previous sputum based surveillance in a tertiary laboratory, with a specialised interest in MABSC culture methodology^16^.

Over the last 2 decades, with the increasing survival of patients with CF, a number of pathogens including MABSC have become of emergent, international importance. Outbreaks of MABSC in non-CF patients have also been reported and, for example, soft tissue, bone and line infections have occurred following the use of contaminated surgical equipment^17^.

To our knowledge there is no directly proven animal-to-human or human-to-human transmission of MABSC. Cross-infection between patients with CF has been suggested however, through elegant, indirect studies combining whole genome sequencing and detailed epidemiology^8^. Recently, Ricketts *et al*.^18^, provided, a case description of suspected human-to-human transmission of *M. kansasii*, involving genetically identical organisms isolated from a husband and wife living in East London. NTM outbreaks have been described following the use of disinfected surgical equipment in patients, with the presumption that this might be due to inadequate cleaning, or be derived from the water supply used to perform the disinfection. The exact source of such MABSC outbreaks has not been precisely identified however^19^.

A breach of “barrier defence” protection against microorganisms is a described risk factor for *M. tuberculosis* (TB)^20^. The stomach is not considered a primary site of infection in these patients, but illustrates this potential niche for mycobacterium and gastric juice can be used for the detection of pulmonary tuberculosis diagnosis in children^13^. No data are available regarding the potential to culture MABSC from gastrostomy to our knowledge however. We have recently shown that gastric juice is an under recognised reservoir of microorganisms relevant to people with CF^9^. Our previous paper investigated the aerodigestive microbiome in people with CF using Next Generation Sequencing but did not study NTM. Our present paper is independent of the first study, using different samples involving the specialised methodology necessary for NTM studies. In this study we investigated the presence of MABSC isolated from sputum, gastric juice and PEG tubes in our regional CF clinic PEG fed patients. The prevalence of MABSC in our combined samples from our PEG fed cohort was 43%; a high rate compared to our latest regional institution study of non-PEG-fed CF patients (16%)^16^, and another recent study (12%)^21^. This may be related to the severity of disease in our PEG-fed patients, however it may also be possible that the presence of a PEG may be associated with MABSC isolation in people with CF.

Our data indicated that MABSC was present in the gastric compartment and was cultured from both gastric juice and PEG tubes. These PEG tubes were removed from patients as part of their routine management and not because of any clinical suspicion of infection. Our findings are in keeping with the known resistance of MABSC organisms to divergent environments including high temperature and low pH^22^.

We are unaware of substantive previous data regarding MABSC culture in non-CF gastric juice. However, we have previously studied the microbiology of 14 gastric juice samples sampled at diagnostic gastroscopy of patients without known lung disease. Our experience showed that In 6 patients whose gastric juice had a pH of 4 or less, bacteria were not cultured. The remaining 8 patients, whose gastric pH was 5 to 8, all had cultures that grew a range of bacteria. These isolates did not include any MABSC or other mycobacteria however^23^,^24^. Our previous preliminary data may therefore suggest some specificity to our finding of MABSC in gastric juice in our PEG fed CF patient group and we conclude that this requires further study. Such studies could explore a novel possibility; that the stomach constitutes a potential reservoir and niche of viable MABSC in PEG fed people with CF. We speculate that this may have a role in failure of eradication treatment in some patients.

Our present study showed an association between MABSC colonisation of gastric juice and sputum in some of our CF patients. Two patients had MABSC in their gastric juice and sputum samples and where MABSC was found concordantly in CF gastric juice and sputum, we demonstrated genetically identical strains by molecular techniques.

Transfer of organisms including MABSC may be possible from lung to stomach through the widely recognised mechanism of cough and swallowing of expectorate. The finding of identical strains of *P. aeruginosa* cultured from the gastric juices and sputa further support the idea that sputum is being swallowed into the stomach. We also found that in a minority of the patients MABSC were isolated from the PEG and gastric juice of patients with MABSC negative sputa. A gastric sample therefore represented the first time that MABSC had been isolated in two patients from our series. These individuals also had negative sputum samples according to the routine clinical microbiology approaches in our centre in the year prior to the GI isolates. Our study cannot indicate that the stomach may be a primary site of infection with MABSC rather than just reflecting swallowing of infected sputum, but this could be a useful area of future research. We have also found that gastric juice and PEG tubes can be culture positive for *P. aeruginosa*, with no *P. aeruginosa* present in the earlier sputum samples. Future studies could therefore test the hypothesis that clinically relevant bacteria including MABSC survive in the gastric compartment independent from respiratory sources, which may have translational importance for the management of people with CF and approaches to attempted eradication of micro-organisms.

An association between NTM lung disease and gastroesophageal disorders has been suggested in previous reports including disease related to *M abscessus*^25^ and reflux and aspiration events have been widely documented in CF patients^26^. Our present study showed a high level of symptoms associated with reflux leaving the oesophagus in our patient cohort, including cough. The validated RSI questionnaire instrument used in this study consists of a series of 9 questions to evaluate extra-oesophageal reflux. All the patients in our cohort colonised with MABSC in their gastric compartment had a high RSI domain score for cough. A single cough can produce a large amount of bioaerosols, which may contribute to infectious disease transmission^27^.

It may therefore be relevant that non-CF patients with pulmonary *Mycobacterium avium* complex (MAC) have a higher incidence of GORD than is reported in the general population^25^,^28^. Moreover, it has been found that the use of regular acid-suppressive medication is associated with an increased risk of MAC pulmonary disease. Acid-suppressive medication is frequently used by CF patients and we have recently contributed to an active debate that PPI usage and raised gastric pH may be associated with bacterial growth^23^. A recent recommendation for management of NTM highlighted the potential importance of PPI as a risk factor^2^. We feel our data is consistent with the need for further research to evaluate the potential roles of GORD and acid suppression, in studies of NTM in CF. A longitudinal study would be needed to address a potential risk of transfer of organisms to the lung from the stomach through mechanisms including cough and recurrent reflux and aspiration. We feel that this should be considered and investigated in CF, and it would be important to also consider the lifelong use of antibiotics in CF patients and their overall effect on microbiomes.

Gastrostomy tube placement is a well-established and tolerated method to augment feeding in patients with CF. The gastrostomy tube may be placed endoscopically, surgically, or fluoroscopically. Foreign body-associated MABSC infections are increasingly recognised now receiving increasing emphasis because of the increased use of indwelling medical devices^29^. The presence of a foreign body is believed to facilitate colonisation by weakening host barrier defence mechanisms^30^. An individual case of perigastrostomy infection caused by *M* abscessus in an immunocompetent patient has been reported^31^.

Because MABSC are resistant to conventional antibiotics and anti-tuberculosis drugs, it is important to recognize this specific group of pathogens, particularly in patients with CF. Early identification may allow timely susceptibility testing, appropriate therapy and PEG management. A strength of our study therefore is that to our knowledge, our findings are the first data showing gastric juice and PEG-tube insertion are a potential niche for MABSC in patients with CF. A weakness of our study is that alongside previous influential studies on MABSC in CF our cohort size was modest and our study is cross sectional. The presence of the PEG allows access to gastric juice without potential commensal sampling from the upper airways but this means that our findings are limited to PEG fed patients with CF. We feel that our preliminary findings might usefully be followed up longitudinally in multi-centre approaches, in a wider range of patients. With clinical concerns raised about the increasing prevalence of MABSC in CF we conclude that such studies are warranted.

## Additional Information

**How to cite this article:** Al-momani, H. *et al*. Nontuberculous mycobacteria in gastrostomy fed patients with cystic fibrosis. *Sci. Rep.* **7**, 46546; doi: 10.1038/srep46546 (2017).

**Publisher's note:** Springer Nature remains neutral with regard to jurisdictional claims in published maps and institutional affiliations.

## Supplementary Material

Supplementary Information

## Figures and Tables

**Table 1:** Demographic data for CF patients.

Patient	Genetics	Age	RSI score	PPI yes/no	Gastric Juice pH	FEV1 (% pred)	BMI	IV days/year	Long-term antibiotic
CF-1	F508del/F508del	26	17	Yes	6	2.0 L (52%)	19.9	22	Azith Inh Coli Inh Tob
CF-2	F508del/F508del	27	20	Yes	2	1.7 L (42%)	23.2	70	Azith Fluclox Inh coli
CF-3	F508del/F508del	20	25	Ranitidine	3	0.8 L (26%)	19.5	28	Azith Fluclox Inh Coli
CF-4	F508del/F508del	24	36	Yes	6	0.76 L (28%)	19	154	Azith Inh Coli
CF-5	F508del/F508del	31	16	Yes	6	0.5 L (12%)	19.1	70	Azith Inh Coli
CF-6	F508del/R117H	22	16	Yes	3	2.7 L (66%)	16.4	14	Fluclox
CF-7	I507del/Arg560Lys	18	13	Yes	2	3.5 L (88%)	19.4	37	Fluclox Inh Coli Inh Tob
CF-8	F508del/R117H	30	14	Yes	6	1.55 L (46%)	17.8	56	Fluclox Inh Coli
CF-9	F508del/F508del	25	17	Yes	2	1.7 L (38%)	15.9	98	Azith Fluclox Inh Tob
CF-10	F508del/G542X	32	NA	No	2	1.15 L (36%)	19.4	112	Azith Inh Coli
CF-11	F508del/F508del	30	19	Yes	6	1.2 L (29%)	19.8	115	Azith Fluclox Inh Coli
CF-1	F508del/G542X	24	15	Yes	2	1.65 (36%)	15.24	197	Azith Inh Coli Inh Tob
CF-13	F508del/rg851Ter	23	22	Yes	6	2.3 (59%)	20.2	56	Azith
CF-14	F508del/F508del	25	24	Yes	4	0.85 L (29%)	17.3	84	Azith Inh Colistin Fluclox
CF-15	F508del/Arg851Ter	25	8	Yes	3	2.1 L (54%)	20.1	56	Doxycycline, Azith
CF-16	F508del/Ile507del	36	NA	Yes	4	0.72 L (17%)	21.9	112	Inh Colistin

Key: Azith = oral azithromycin long-term. Fluclox = oral flucloxacillin long-term. Inh Coli = inhaled colistin (nebulised or inhaler). Inh Tob = inhaled tobramycin (nebulised or inhaler). RSI score = Reflux symptom index score, <13 normal. NA = not available, IV days/year = Number of days per year that CF patient treated with intravenous antibiotics.

**Table 2:** Patient details related to chronic colonisation of NTM.

	Gastric juice	sputum	PEG	NTM status
CF-1	*M massiliense*	No MABSC isolated	No PEG*	no previous MABSC
CF-3	*M massiliense*	*M massiliense*	No PEG	+ve for *M massiliense* since 6-12-06
CF-4	No MABSC isolated	*M abscessus sub sp abscessus*	*M abscessus sub sp abscessus*	+ve for *M abscessus* 10-7-08
CF-6	No MABSC isolated	*M abscessus sub sp abscessus*	No MABSC isolated	+ve for *M abscessus* 9-1-09
CF-9	No MABSC isolated	*M massiliense*	No PEG	no previous MABSC
CF-13	*M massiliense*	*M massiliense*	No PEG	Grew *M chelonae* once only
CF-14	No MABSC isolated	No MABSC isolated	M abscessus sub sp abscessus Mycobacterium bolletii	no previous MABSC

Key: * = No PEG tube isolated from the patients.

## References

[b1] Thomson R. M. Changing epidemiology of pulmonary nontuberculous mycobacteria infections. Emerg. Infect. Dis. 16, 1576–1584 (2010). 20875283 10.3201/eid1610.091201 PMC3294381

[b2] Floto R. A. . US Cystic Fibrosis Foundation and European Cystic Fibrosis Society consensus recommendations for the management of non-tuberculous mycobacteria in individuals with cystic fibrosis. Thorax 71, i1–i22 (2016). 26666259 10.1136/thoraxjnl-2015-207360 PMC4717371

[b3] Esther C. R., Esserman D. A., Gilligan P., Kerr A. & Noone P. G. Chronic Mycobacterium abscessus infection and lung function decline in cystic fibrosis. Journal of Cystic Fibrosis 9, 117–123 (2010). 20071249 10.1016/j.jcf.2009.12.001 PMC3837580

[b4] Orens J. B. . International guidelines for the selection of lung transplant candidates: 2006 update—a consensus report from the Pulmonary Scientific Council of the International Society for Heart and Lung Transplantation. The Journal of heart and lung transplantation 25, 745–755 (2006). 16818116 10.1016/j.healun.2006.03.011

[b5] Jarand J. . Clinical and microbiologic outcomes in patients receiving treatment for Mycobacterium abscessus pulmonary disease. Clin. Infect. Dis. 52, 565–571 (2011). 21292659 10.1093/cid/ciq237

[b6] Adékambi T. & Drancourt M. Dissection of phylogenetic relationships among 19 rapidly growing Mycobacterium species by 16S rRNA, hsp65, sodA, recA and rpoB gene sequencing. International journal of systematic and evolutionary microbiology 54, 2095–2105 (2004). 15545441 10.1099/ijs.0.63094-0

[b7] Harris K. A. . Molecular fingerprinting of Mycobacterium abscessus strains in a cohort of paediatric Cystic Fibrosis patients. J. Clin. Microbiol., JCM. 00155–00112 (2012). 10.1128/JCM.00155-12 PMC3347134 22403419

[b8] Bryant J. M. . Whole-genome sequencing to identify transmission of Mycobacterium abscessus between patients with cystic fibrosis: a retrospective cohort study. The Lancet 381, 1551–1560 (2013). 10.1016/S0140-6736(13)60632-7 PMC3664974 23541540

[b9] Al-Momani H. . Microbiological profiles of sputum and gastric juice aspirates in Cystic Fibrosis patients. Sci. Rep. 6, 26985 (2016). 27245316 10.1038/srep26985 PMC4887896

[b10] Blondeau K. . Gastro-oesophageal reflux and aspiration of gastric contents in adult patients with cystic fibrosis. Gut 57, 1049–1055 (2008). 18372497 10.1136/gut.2007.146134

[b11] Lobato M. N., Loeffler A. M., Furst K., Cole B. & Hopewell P. C. Detection of Mycobacterium tuberculosis in gastric aspirates collected from children: hospitalization is not necessary. Pediatrics 102, e40–e40 (1998). 9755277 10.1542/peds.102.4.e40

[b12] Hatherill M. . Induced sputum or gastric lavage for community-based diagnosis of childhood pulmonary tuberculosis? Arch. Dis. Child. 94, 195–201 (2009). 18829621 10.1136/adc.2007.136929

[b13] Abadco D. L. & Steiner P. Gastric lavage is better than bronchoalveolar lavage for isolation of Mycobacterium tuberculosis in childhood pulmonary tuberculosis. The Pediatric infectious disease journal 11, 735–738 (1992). 1448314 10.1097/00006454-199209000-00013

[b14] Belafsky P. C., Postma G. N. & Koufman J. A. Validity and reliability of the reflux symptom index (RSI). J. Voice 16, 274–277 (2002). 12150380 10.1016/s0892-1997(02)00097-8

[b15] Blauwendraat C., Dixon G. L. J., Hartley J. C., Foweraker J. & Harris K. A. The use of a two-gene sequencing approach to accurately distinguish between the species within the Mycobacterium abscessus complex and Mycobacterium chelonae. Eur. J. Clin. Microbiol. Infect. Dis. 31, 1847–1853 (2012). 22222989 10.1007/s10096-011-1510-9

[b16] Preece C. L. . A novel culture medium for isolation of rapidly-growing mycobacteria from the sputum of patients with cystic fibrosis. Journal of Cystic Fibrosis 15, 186–191 (2016). 26002312 10.1016/j.jcf.2015.05.002

[b17] Wallace R. J. Jr., Brown B. A. & Griffith D. E. Nosocomial outbreaks/pseudo outbreaks caused by nontuberculous mycobacteria. Annual Reviews in Microbiology 52, 453–490 (1998). 10.1146/annurev.micro.52.1.453 9891805

[b18] Ricketts W. M., O’Shaughnessy T. C. & van Ingen J. Human-to-human transmission of Mycobacterium kansasii or victims of a shared source? Eur. Respir. J. 44, 1085–1087 (2014). 24969652 10.1183/09031936.00066614

[b19] Griffith D. E. . An official ATS/IDSA statement: diagnosis, treatment, and prevention of nontuberculous mycobacterial diseases. Am. J. Respir. Crit. Care Med. 175, 367–416 (2007). 17277290 10.1164/rccm.200604-571ST

[b20] Snider D. E. Tuberculosis and gastrectomy. CHEST Journal 87, 414–415 (1985). 10.1378/chest.87.4.414 3979126

[b21] Adjemian J., Olivier K. N. & Prevots D. R. Nontuberculous mycobacteria among patients with cystic fibrosis in the United States. Screening practices and environmental risk. Am. J. Respir. Crit. Care Med. 190, 581–586 (2014). 25068291 10.1164/rccm.201405-0884OC PMC4214089

[b22] Bodmer T., Miltner E. & Bermudez L. E. Mycobacterium avium resists exposure to the acidic conditions of the stomach. FEMS Microbiol. Lett. 182, 45–49 (2000). 10612729 10.1111/j.1574-6968.2000.tb08871.x

[b23] Jones R., Pearson J. & Ward C. Functional Dyspepsia. N Engl J Med 374, 895–889 (2016). 10.1056/NEJMc1515497 26962925

[b24] Al-momani H. . Microbiological profiles of sputum and gastric juice aspirates in Cystic Fibrosis patients. Scientific reports 6 (2016). 10.1038/srep26985 PMC4887896 27245316

[b25] Koh W.-J. . Prevalence of gastroesophageal reflux disease in patients with nontuberculous mycobacterial lung disease. CHEST Journal 131, 1825–1830 (2007). 10.1378/chest.06-2280 17400680

[b26] Brodzicki J., Trawińska M. & Korzon M. Frequency, consequences and pharmacological treatment of gastroesophageal reflux in children with cystic fibrosis. Med. Sci. Monit. 8, CR529–CR537 (2002). 12118204

[b27] Fennelly K. P. . Variability of infectious aerosols produced during coughing by patients with pulmonary tuberculosis. Am. J. Respir. Crit. Care Med. 186, 450–457 (2012). 22798319 10.1164/rccm.201203-0444OC PMC3443801

[b28] Thomson R. M., Armstrong J. G. & Looke D. F. Gastroesophageal reflux disease, acid suppression, and Mycobacterium avium complex pulmonary disease. CHEST Journal 131, 1166–1172 (2007). 10.1378/chest.06-1906 17426224

[b29] Trupiano J. K. . Mastitis due to Mycobacterium abscessus after body piercing. Clin. Infect. Dis. 33, 131–134 (2001). 11389508 10.1086/320885

[b30] Linmans J. J., Stokroos R. J. & Linssen C. F. M. Mycobacterium abscessus, an uncommon cause of chronic otitis media: a case report and literature review. Archives of Otolaryngology–Head & Neck Surgery 134, 1004–1006 (2008). 18794448 10.1001/archotol.134.9.1004

[b31] Chiu H.-Y., Liu K.-L. & Liao Y.-H. Perigastrostomy infection caused by Mycobacterium abscessus in an immunocompetent patient. Acta Derm. Venereol. 90, 100–101 (2010). 20107743 10.2340/00015555-0784

