# Supplementary material for: Nontuberculous mycobacteria in gastrostomy fed patients with cystic fibrosis
Source: Sci Rep. 2017 Apr 24;7:46546. doi: 10.1038/srep46546 (PMC5402269; doi:10.1038/srep46546)
Supplement: Supplementary Information [file srep46546-s1.pdf]

Nontuberculous mycobacteria in gastrostomy fed patients with cystic fibrosis

H Al-momani, A Perry, R Jones, S Bourke, S Doe, J Perry, A Anderson, T Forrest, I Forrest, M Griffin, M Brodlie, J Pearson, C Ward

Supplemental Material

E-Table 1: CF gastric juice microbiological cultures

Gastric juice 1	*Candida glabrata, Enterococcus faecium, Lactobacillus paracasi, Saccharomyces cerevisiae* and *Mycobacterium abscessus* subsp *massiliense*
Gastric juice 2	*Candida lustania* and *Candida krusei*
Gastric juice 3	*Candida albicans* and *Mycobacterium abscessus* subsp *massiliense*
Gastric juice 4	*Candida albicans, Candida krusie* and *Lactobacillius fermentum.*
Gastric juice 5	*Streptococcus mitis, lactobacillus rhamnosus, Saccharomyces cerevisiae, Achromobacter xylosoxidans* and *Pseudomonas aeruginosa*
Gastric juice 6	*Candida albicans, Candida glabrata* and *Lactobacillius plantarum*
Gastric juice 7	*Candida albicans* and *Lactobacillus gasseri*
Gastric juice 8	*Candida krusei, Candida glabrata* and *Pseudomonas aeruginosa*
Gastric juice 9	*Candida albicans, Candida dubliniensis* and *Lactobacillius gasseri*
Gastric juice 10	*Candida glabrata* and *Staphylococcus hominis*
Gastric juice 11	*Candida albicans, Candida parapsilosis, Hafnia alvei, Enterococcus faecalis, Serratia fonticola* and *Raoultella sp*
Gastric juice 12	*Candida albicans, Candida glabrata* and *Candida krusei*
Gastric juice 13	*Candida albicans, Candida glabrata* and *Mycobacterium abscessus* subsp m*assiliense*
Gastric juice 14	*Candida albicans, Streptococcus salivarius, Streptococcus mitis, Rothia mucilaginosa, Achromobacter xylosoxidans, Pseudomonas aeruginosa* and *Neisseria sp*
Gastric juice 15	*Candida glabrata* and *Exophiala dermatitidis*
Gastric juice 16	*Candida albicans, Escherichia coli* and *Pseudomonas aeruginosa*

E-Table 2: Sputum sample microbiology

Sputum 1	*Candida albicans, Streptococcus anginosus, Streptococcus mitis* and *Rothia dentocariosa*
Sputum 2	*Aspergillus fumigatus, Streptococcus mitis, Streptococcus parasanguinis, Streptococcus salivarius, Rothia mucilaginosa* and *Achromobacter xylosoxidans*
Sputum 3	*Streptococcus oralis, Rothia mucilaginosa Pseudomonas aeruginosa* and *Mycobacterium abscessus* subsp *massiliense*
Sputum 4	*Candida albicans, Aspergillus fumigatus, Rothia mucilaginosa, Rothia dentocariosa, Streptococcus mitis, Streptococcus sanguinis, Streptococcus oralis and Mycobacterium abscessus* subsp. *abscessus*
Sputum 5	*Candida albicans, Rothia mucilaginosa, Rothia dentocariosa, Streptococcus mitis and Streptococcus oralis*
Sputum 6	*Candida albicans, Streptococcus mitis, Rothia mucilaginosa, Neisseria meningitidis, Haemophilus influenzae* and *Mycobacterium abscessus* subsp. *abscessus*
Sputum 7	*Streptococcus mitis* and *Rothia mucilaginosa*
Sputum 8	*Streptococcus oralis, Rothia, Haemophilus parainfluenzae* and *Pseudomonas aeruginosa*
Sputum 9	*Rothia mucilaginosa, Streptococcus sanguinis, Streptococcus mitis, Pseudomonas aeruginosa, Burkholderia multivorans, Actinomyces oris, Penicillium* and *Mycobacterium abscessus* subsp *massiliense*
Sputum 10	*Candida albicans, Streptococcus mitis* and *Rothia mucilaginosa*
Sputum 11	*Pseudomonas aeruginosa*
Sputum 12	*Candida albicans, Achromobacter xyosoxidans* and *Pseudomonas aeruginosa*
Sputum 13	*Streptococcus mitis, Stenophomonas maltophilia, Staphylococcus aureus, Cardiobacterium hominis, maltophilia, Veillonella parvula* and *Mycobacterium abscessus* subsp *massiliense*
Sputum 14	*Streptococcus oralis, Streptococcus mitis, Streptococcus sanguinis, Rothia dentocariosa, Rothia aeria, Klebsiell oxytoca, Neisseria flavescens, Achromobacter xyxlosoxidans* and *Pseudomonas aeruginosa*
Sputum 15	*Staphylococcus warneri, Neisseria sp, Streptococcus parasanguinis,* Stenotrophomonas *maltophilia* and *Exophiala dermatitidis*
Sputum 16	*Aspergillus fumigatus, Escherichia coli* and *Pseudomonas aeruginosa*

E-Table 3: PEG-s sample microbiology

PEG 4	*Candida albicans, Candida krusie, Candida glabrata Candida parapsilosis Lactobacillius fermentum, Enterococcus faecium* and *Mycobacterium abscessus sub sp abscessus*
PEG 5	*Candida albicans, Candida parapsilosis, Saccharomyces cerevisiae, Staphylococcus epidermidis* and *Pseudomonas aeruginosa*
PEG 6	*Enterobacter cloacae, Stenotrophomonas maltophilia* and *Pseudomonas aeruginosa*
PEG 14	*Candida albicans, Streptococcus oralis, Mycobacterium abscessus sub sp abscessus Mycobacterium bolletii* and *Aspergillus fumigatus*
PEG 15	*Candida glabrata Candida parapsilosis Staphylococcus epidermidis* and *Lactobacillus paracasei*
